# Does cultural capital contribute to educational inequalities in food consumption in the Netherlands? A cross-sectional analysis of the GLOBE-2011 survey

**DOI:** 10.1186/s12939-018-0884-z

**Published:** 2018-11-15

**Authors:** Carlijn B. M. Kamphuis, Joost Oude Groeniger, Frank J. van Lenthe

**Affiliations:** 1 Department of Public Health, Erasmus University Medical Centre, PO Box 2040, 3000 CA Rotterdam, The Netherlands; 2 Department of Human Geography and Spatial Planning, Utrecht University, PO Box 80140, 3508 TC Utrecht, The Netherlands; 3 Department of Interdisciplinary Social Science, Utrecht University, PO Box 80140, 3508 TC Utrecht, The Netherlands

**Keywords:** Cultural capital, Social capital, Economic capital, Bourdieu, Educational inequalities, Socioeconomic position, Healthy food consumption

## Abstract

**Background:**

The importance of culture for food consumption is widely acknowledged, as well as the fact that culture-based resources (“cultural capital”) differ between educational groups. Since current explanations for educational inequalities in healthy and unhealthy food consumption (e.g. economic capital, social capital) are unable to fully explain this gradient, we aim to investigate a new explanation for educational inequalities in healthy food consumption, i.e. the role of cultural capital.

**Methods:**

Data were obtained cross-sectionally by a postal survey among participants of the GLOBE study in the Netherlands in 2011 (*N* = 2953; response 67.1%). The survey measured respondents’ highest attained educational level, food-related cultural capital (institutionalised, objectivised and incorporated cultural capital), economic capital (e.g. home ownership, financial strain), social capital (e.g. social support, health-related social leverage, interpersonal relationships), and frequency of consumption of healthy and unhealthy food products. Two general outcomes (overall healthy food consumption, and overall unhealthy food consumption), and seven specific food consumption outcomes were constructed, and prevalence ratios (PR) were estimated in Poisson regression models with robust variance.

**Results:**

Cultural capital was significantly associated with all food outcomes, also when social and economic capital were taken into account. Those with low levels of cultural capital were more likely to have a lower overall healthy food consumption (PR 1.35, 95% CI 1.22–1.49), a lower consumption of whole wheat bread (PR 1.21, 95% CI 1.05–1.38), vegetables (PR 1.55, 95% CI 1.40–1.71), and meat-substitutes and fish (PR 1.74, 95% CI 1.53–1.97), and a higher consumption of fried food (PR 1.59, 95% CI 1.31–1.93). Social capital was positively associated with overall healthy food consumption, whole wheat bread consumption, and the consumption of fish and meat-substitutes, and economic capital with none of the outcomes. The PR of the lowest educational group to have a low overall healthy food consumption decreased from 1.48 (95% CI 1.28–1.73) to 1.22 (95% CI 1.04–1.43) when cultural, social and economic capital were taken into account.

**Conclusions:**

Cultural capital contributed to the explanation of educational inequalities in food consumption in The Netherlands, over and above economic and social capital. The socialisation processes through which cultural capital is acquired could offer new entry-points for the promotion of healthy food consumption among low educational groups.

**Electronic supplementary material:**

The online version of this article (10.1186/s12939-018-0884-z) contains supplementary material, which is available to authorized users.

## Introduction

Studies consistently find a socioeconomic gradient in healthy dietary intakes [1–6]. However, interventions to encourage healthy food consumption have only had small effects, and if so, particularly among high socioeconomic groups [7, 8]. Therefore, there is a high need to identify relevant determinants of healthy food consumption in order to find entry points for developing interventions that may increase healthier food consumption, especially among low socioeconomic groups.

Previous studies have identified explanatory factors that partly explain socioeconomic inequalities in diet. Economic resources, such as an adequate food budget, are typically connected to an individual’s socioeconomic position, and to healthy food intakes, as lower-quality diets generally cost less per calorie [1, 9, 10]. Also, social resources, measured through membership in support-providing networks, perceived social support, or perceived social norms, have shown to be associated with healthy food intakes [11, 12], although their contribution to socioeconomic differences in healthy food consumption is less clear [1, 13]. As measures of economic and social resources cannot fully explain the socioeconomic gradient in healthy food consumption, recently, studies have appeared taking a different angle. These studies have linked cultural resources to health inequalities [14, 15], and have argued that culture-based activities, knowledge and perceptions present a unique form of health-relevant ‘capital’.

Culture can be defined as the culture-based resources that shape and influence people’s habits, values, norms, knowledge and preferences, acquired mostly through social learning [14]. Learning conditions vary across socioeconomic groups and milieus, and so culture does as well [14]. Culture, further, is well-known for its important nfluence on food consumption, as it determines what people consider to be acceptable and preferable foods, and what the amount and combinations of food they choose [16, 17]. Although some research has emerged over the last few years [18–20], empirical evidence for the role of cultural factors for the explanation of socioeconomic inequalities in food consumption is still limited.

Among the most influential studies regarding the role of culture for daily practices is the work of the French sociologist Pierre Bourdieu (1930–2003) [21]. High socioeconomic groups, over their life courses, acquire more capital and ‘use’ this to develop a taste for specific forms of music, lecture, leisure activities, and foods. Bourdieu defines three types of capital that play a role in this process, namely cultural, economic, and social capital. Cultural capital is a non-material resource that accumulates throughout the life course, acquired through education and life-long socialisation, and includes “the distinctive forms of knowledge and ability that people acquire [...] from their training in the cultural disciplines” [21]. Through available cultural capital in the family, one is more inclined to ‘inherit’ cultural resources that can be mobilised to accumulate incorporated cultural capital [22]. Cultural capital emerges in three different states: incorporated cultural capital (e.g. values, skills, cultural participation), objectivised cultural capital (e.g. books, tools) and institutionalised cultural capital (e.g. educational degrees, professional titles) [21]. Lareau and Weininger ([23], p. 156) refer to incorporated cultural capital as “the legitimate cultural attitudes, preferences and behaviours […] that are internalized during the socialization process”. Incorporated cultural capital, “the form of long-lasting dispositions of the mind and the body”, entails socialisation, personal effort, and time investment ([22], p. 47). In line with reflections by Abel [14], we expect incorporated cultural capital to be more important for educational inequalities in food choices than institutionalised and objectivised cultural capital. Also, incorporated cultural capital has the largest potential to be on the causal chain between socioeconomic position and healthy food choices. It is not possible to convey incorporated cultural capital to someone else, as would be possible with economic capital or objectivised cultural capital.

Besides cultural capital, Bourdieu also acknowledges the importance of economic and social capital. Economic capital comprises all sources of income (including wealth), as well as the security of having a reliable income. Social capital is defined by Bourdieu as “the aggregate of the actual or potential resources which are linked to possession of a durable network of more or less institutionalized relationships of mutual acquaintance or recognition”. Economic, social and cultural capital are correlated and feed on each other. The different forms of capital can be converted as, for example, personal savings (economic capital) can be used to pay for advanced education (cultural capital) [14]. The roles of economic and social capital for inequalities in health and health-behaviours have been studied rather extensively, whereas the role of cultural capital is largely unknown. The aim of this study is to investigate whether cultural capital contributes to the explanation of socioeconomic inequalities in food consumption among adults, over and above social and economic capital.

## Methods

Self-reported data were collected by means of a large-scale postal survey in 2011, administered as a new wave of data collection for the longitudinal GLOBE study [24]. The research was conducted according to the Declaration of Helsinki, and informed consent was obtained from all subjects. No formal approval of the medical ethics committee of the Erasmus University Medical Centre was required for the study. The use of personal data in the GLOBE study is in compliance with the Dutch Personal Data Protection Act and the Municipal Database Act, and has been registered with the Dutch Data Protection Authority (number 1248943).

Of the respondents to the previous GLOBE survey in 2004, which formed a stratified sample of the 25–75 years old population in the city of Eindhoven and surrounding cities in 2004 (*N* = 4784), *n* = 249 had died, *n* = 76 had emigrated, and *n* = 14 were lost to follow-up (i.e. no correct address information available), which resulted in a sample of *N* = 4437 that was sent the 2011-survey. For the total of 2983 respondents that returned the survey (response 67.2%), missing values for sex (*n* = 21), age (*n* = 24), and educational level (*n* = 172) could largely be replaced by information from the 2004-questionnaire, resulting in only one case with a missing value for age and 29 cases with missing values for educational level. These 30 respondents were excluded from the analysis, resulting in an analytic sample of *N* = 2953.

### Educational level as indicator of socioeconomic position, and demographic variables

Educational level has traditionally been the most important indicator of social stratification in Dutch society [25, 26]. It is also an appropriate indicator of socioeconomic position to classify both men and women, in contrast to occupational level and income level (as women are more likely than men to not have a paid job) [27]. Respondents indicated their highest attained educational level, which was classified according to the International Standard Classification of Education (ISCED): 1 – primary education (ISCED 0–1), 2 – lower secondary education (ISCED 2), 3 – upper secondary education (ISCED 3–4), 4 – tertiary education (ISCED 5–7). All analyses were adjusted for marital status (married, single/divorced/widowed), ethnic background (native Dutch, other), age and sex.

### Food consumption

With a Food Frequency Questionnaire (based on existing questionnaires [28–30]) that was part of the GLOBE 2011-survey, we obtained self-reported information on the frequency with which 26 specific food groups were consumed. Participants indicated the number of days per week a certain food product was consumed. This number was converted to an indicator for ‘average daily frequency’ by the following formula [31]: never: 0; less than once a week: 0.10; 1–2 days per week: 0.20; 3–4 days per week: 0.50; 5–6 days per week: 0.80; every day: 1.

A ‘healthy foods’ score was constructed as the sum of the consumption of fruit, cooked vegetables, raw vegetables, whole wheat bread, skimmed milk, low fat cheese, chicken, fish, and meat-substitutes (like tofu). To calculate this score, the average daily frequencies (ranging from 0 till 1, as detailed above) for each of these products were summed. Similarly, an ‘unhealthy foods’ score was constructed as the sum of the frequencies of consumption of fried food, candy, white bread, soft drinks, whole milk, high fat cheese, and red meat (beef, pork, lamb, mince, and burgers). These indices were considered useful as general measures of healthy and unhealthy eating [31], since the specific food products included in these measures are also recognized as typically healthy or unhealthy by international authorities, such as the American Heart Foundation, the British Nutrition Foundation and the Netherlands Nutrition Centre [32–34]. For detailed analyses, seven specific food outcomes were analysed, representing four typically healthy food groups (whole wheat bread, fruits, vegetables, and meat-substitutes & fish) and three typically unhealthy food groups (red meat (beef, pork, mince, and burgers), fried food, and soft drinks). Means and standard deviations for the raw daily frequency scores by educational level are presented in Additional file 1. As these scores had skewed distributions, the variables were dichotomised with the median as cut-off point, i.e. half of the sample was categorised as having a ‘low’ consumption, and half of the sample as ‘high’ consumption.

### Cultural, social and economic capital

We generated composite variables of cultural, social and economic capital based on the scores of the constituent items chosen for capturing each type of capital. Table 1 lists the variables for cultural, social and economic capital, their categorisation for the analyses, and the items that comprised each variable. To construct the variables, several items were combined by means of a factor analyses or a mean score, and these were further divided in tertiles. A detailed description of the measurement and construction of each variable is available in Additional file 2.

**Table 1:** Measurement and construction of the variables for cultural, social and economic capital

Variables	Measurement in the survey	Categorisation of the variable for the analyses
Family institutionalised cultural capital	Educational level of the respondent’s father, mother and partner	1 = low, 2 = mid, 3 = high (tertiles of mean score)
Objectivised cultural capital	Number of cooking-related possessions, i.e. a stove, cook book(s), set of knives, kitchen scale, and juicer (yes/no)	1 = low, 2 = mid, 3 = high (tertiles of sum score)
Incorporated cultural capital	Participation, cooking skills, grocery shopping skills, information seeking and processing skills, nutrition knowledge	1 = low, 2 = mid, 3 = high (tertiles of mean score)
Total cultural capital	Mean score of the variables for family institutionalised, objectivised, and total incorporated cultural capital	1 = low, 2 = mid, 3 = high (tertiles of mean score)
Total social capital	Social support, health-related social leverage, interpersonal relationship network, social participation, perceptions of trust, perceived neighbourhood social capital	1 = low, 2 = mid, 3 = high (tertiles of mean score)
Total economic capital	Household equivalent income, home ownership, crowding, financial strain	1 = low, 2 = mid, 3 = high (tertiles of mean score)

Note: Detailed information on measurement and construction of the variables is available in Additional file 2. Information on the development of the cultural capital questionnaire is described elsewhere [5].

In short, we used an existing questionnaire to measure the three forms of cultural capital in relation to food [5]. This questionnaire has been developed based on a systematic review to identify existing indicators of cultural capital. The indicators that have been used most often in the literature, were translated to food-related indicators [5]. Objectivised cultural capital was consistently measured in the literature by cultural possessions (e.g. art, books) and we translated this to a list of possessions related to food choice behaviour. In the survey, participants reported whether they owned a list of cooking-related possessions, e.g. cook books, kitchen scale, juicer. Scores were summed and the sum score was divided in tertiles (low, medium, high objectivised cultural capital). Incorporated cultural capital was operationalised by items on participation, cooking skills, grocery shopping skills, information seeking skills, and nutrition knowledge. Scores on the different items were summed and the sum score was divided in tertiles (low, medium, high incorporated cultural capital). Institutionalised cultural capital appeared to be most often operationalised by educational level of the respondent [5]. However, since we were interested in understanding educational inequalities (i.e. we used own education level as indicator of socioeconomic position), we focussed on the socialisation processes in which acquisition of cultural capital takes place, and therefore used educational level of the father, mother and partner of the respondent as indicators of institutionalised cultural capital. Scores were summed and the sum score was divided in tertiles (low, medium, high institutionalised cultural capital). The three types of cultural capital were analysed as separate variables. A mean score of these three variables was used as indicator of total cultural capital, which was divided into tertiles (low, medium, high total cultural capital).

For social capital, indicators of six dimensions of social capital (e.g. social support, health-related social leverage, interpersonal relationship network) [35] were combined in one score for total social capital, which was divided into tertiles (low, medium, high social capital). Economic capital was measured by four commonly used indicators (e.g. home ownership, financial strain) [36] and their mean score was divided into tertiles (low, medium, high economic capital).

### Statistical analyses

Statistical analyses were conducted in SPSS 20.0. Since our outcomes are not rare (i.e. greater than 10%), we follow recommendations to calculate prevalence ratios (PR’s) as measure of association, instead of odds ratios (OR’s), as the interpretation of the OR is difficult and often mistakenly interpreted as PR [37, 38]. In Poisson regression models with robust variance, PR’s with 95% confidence intervals were calculated for each of the outcomes by educational level, adjusted for age, sex, ethnicity, and marital status. Further, PR’s with 95% confidence intervals were calculated for each of the outcomes by each type of capital in separate models, adjusted for educational level, age, sex, ethnicity, and marital status level. In multivariate Poisson regression models, we included all capital variables simultaneously to observe which types of capital remained significantly associated with food consumption when mutually adjusted, and to observe whether the PR’s for the low educational group would attenuate after inclusion of the capital variables, compared to the model with only confounders. This reduction in PR’s was interpreted as the contribution of the capital variables to the explanation of educational inequalities in food consumption.

## Results

The mean age of the sample was 56.4 years (SD 13.0) and 56.7% was female (Table 2). In general, educational inequalities in healthy food consumption were larger than those in unhealthy food consumption (Table 3). Low educated were more likely to report a low overall healthy food consumption (PR 1.48, 95% CI 1.28–1.73), low whole wheat bread consumption (PR 1.38, 95% CI 1.08–1.76), low vegetable consumption (PR 1.46, 95% CI 1.23–1.73) and a low consumption of meat-substitutes and fish (PR 1.66, 95% CI 1.37–2.02) than high educated. Regarding unhealthy food outcomes, low educational groups were about twice as likely to have a high fried food consumption (PR 2.03, 95% CI 1.44–2.86), but no significant inequalities in overall unhealthy food, red meat, or soft drink consumption were observed. Outcomes for which no educational inequalities were found, were not further analysed in multivariate models.

**Table 2:** Study sample characteristics: demographic factors, and cultural, social and economic capital by educational level

		Educational level			
	Total	1-low	2-midlow	3-midhigh	4-high
	(*N* = 2953)^a^	(*n* = 263)^a^	(*n* = 1041)^a^	(*n* = 678)^a^	(*n* = 971)^a^
	%^b^	%^b^	%^b^	%^b^	%^b^
Sex					
Men	43.3	41.4	32.9	44.5	51.8
Women	56.7	58.6	67.1	55.5	48.2
Marital status					
Married, registered partnership	74.9	63.2	72.4	78.6	76.5
Single, divorced, widowed	24.6	34.6	27.2	21.0	23.2
Missing	0.5	2.2	0.4	0.4	0.3
Ethnic background^c^					
Dutch	84.5	64.9	85.4	87.5	84.9
Other	10.4	17.8	8.5	10.2	10.9
Missing	5.1	17.3	6.0	2.3	4.2
Age, mean, in years (SD)	56.4 (13.0)	66.0 (12.3)	61.8 (10.7)	52.9 (12.2)	52.6 (12.9)
Total cultural capital					
Low	39.1	81.6	53.6	38.3	19.6
Mid	24.4	7.6	27.0	24.2	25.1
High	36.4	10.3	19.3	37.5	55.2
Missing	0.1	0.5	0.1	0.0	0.1
Institutionalised cultural capital					
Low	39.4	53.5	60.9	36.2	20.3
Mid	20.1	1.6	11.4	26.8	26.0
High	30.3	9.2	12.5	30.1	49.8
Missing	10.2	35.7	15.1	6.9	4.0
Objectivised cultural capital					
Low	19.1	52.7	22.2	16.2	12.6
Mid	23.7	17.2	25.8	24.8	22.1
High	56.0	24.2	50.	58.2	64.7
Missing	1.3	5.9	1.6	0.8	0.6
Incorporated cultural capital					
Low	29.9	59.7	35.5	28.4	21.0
Mid	38.4	26.9	42.1	40.4	35.7
High	31.5	12.9	22.2	31.3	43.1
Missing	0.2	0.5	0.2	0.0	0.3
Total social capital					
Low	32.0	53.0	35.0	31.4	26.2
Mid	32.6	28.6	34.0	31.3	33.1
High	35.4	18.4	31.0	37.0	40.8
Missing	0.0	0.0	0.0	0.0	0.0
Total economic capital					
Low	36.5	80.0	50.2	36.7	16.8
Mid	35.3	14.6	31.4	36.2	41.6
High	27.9	5.4	17.8	27.1	41.3
Missing	0.3	0.0	0.5	0.0	0.3

^a^All numbers (*N*) are unweighted and reflect the actual numbers of participants in the dataset

^b^All percentages (%) are weighted and thereby represent the prevalence rates as they existed in the population of Eindhoven of 2004, which is the source population. The weight factors were calculated from the distribution of the characteristics in a random sample drawn from the municipal registry in Eindhoven, October 2004

^c^Dutch: both parents of the respondent were born in the Netherlands (definition by Statistics Netherlands). Other: at least one parent of the respondent was not born in the Netherlands

**Table 3:** Separate poisson regression models for educational level, total cultural capital, three specific types of cultural capital, total social capital, and total economic capital in their association with food consumption (adjusted for confounders^a^)

	*Sum scores*				*Specific food outcomes*													
	Low overall healthy food consumption		High overall unhealthy food consumption		Low whole wheat bread		Low fruit		Low vegetables		Low fish/meat substitute		High fried food		High red meat		High soft drink	
	*(n = 2947)* ^b^		*(n = 2938)* ^b^		*(n = 2900)* ^b^		*(n = 2902)* ^b^		*(n = 2934)* ^b^		*(n = 2822)* ^b^		*(n = 2853)* ^b^		*(n = 2906)* ^b^		*(n = 2747)* ^b^	
	PR	(95% CI)	PR	(95% CI)	PR	(95% CI)	PR	(95% CI)	PR	(95% CI)	PR	(95% CI)	PR	(95% CI)	PR	(95% CI)	PR	(95% CI)
Educational level																		
1 High	1.00		1.00		1.00		1.00		1.00		1.00		1.00		1.00		1.00	
2	1.18***	(1.07–1.30)	1.01	(0.92–1.12)	1.32***	(1.16–1.49)	1.09*	(1.01–1.17)	1.32***	(1.23–1.73)	1.33***	(1.18–1.50)	1.59***	(1.33–1.90)	1.03	(0.94–1.14)	1.00	(0.91–1.10)
3	1.24***	(1.12–1.37)	1.00	(0.90–1.12)	1.40***	(1.22–1.61)	1.04	(0.95–1.14)	1.38***	(1.25–1.52)	1.36***	(1.21–1.54)	1.60***	(1.30–1.97)	0.93	(0.83–1.03)	1.05	(0.95–1.16)
4 Low	1.48***	(1.28–1.73)	0.88	(0.71–1.10)	1.38**	(1.08–1.76)	1.12	(0.93–1.34)	1.46***	(1.23–1.73)	1.66***	(1.37–2.02)	2.03***	(1.44–2.86)	1.11	(0.92–1.35)	1.16	(0.96–1.41)
Total cultural capital																		
High	1.00		1.00		1.00		1.00		1.00		1.00		1.00		1.00		1.00	
Mid	1.24***	(1.12–.139)	1.09	(0.97–1.22)	1.14	(0.99–1.31)	1.13*	(1.03–1.24)	1.13**	(1.03–1.24)	1.33***	(1.16–1.52)	1.29*	(1.05–1.58)	0.96	(0.86–1.07)	1.14**	(1.02–1.27)
Low	1.38***	(1.25–1.53)	1.19**	(1.07–1.32)	1.27***	(1.11–1.45)	1.28***	(1.18–1.40)	1.28***	(1.18–1.40)	1.77***	(1.56–2.00)	1.58***	(1.31–1.91)	1.06	(0.96–1.18)	1.30***	(1.18–1.44)
Institutionalised cultural capital																		
High	1.00		1.00		1.00		1.00		1.00		1.00		1.00		1.00		1.00	
Mid	1.11	(0.99–1.25)	0.97	(0.86–1.09)	1.03	(0.89–1.20)	1.14**	(1.05–1.25)	1.10	(0.97–1.23)	0.98	(0.85–1.13)	1.24	(1.00–1.53)	1.07	(0.96–1.19)	1.05	(0.95–1.17)
Low	1.20***	(1.08–1.34)	0.95	(0.85–1.07)	1.26***	(1.09–1.45)	1.12*	(1.02–1.23)	1.36***	(1.23–1.52)	1.21**	(1.07–1.38)	1.31*	(1.07–1.62)	1.00	(0.90–1.11)	1.09	(0.98–1.21)
Objectivised cultural capital																		
High	1.00		1.00		1.00		1.00		1.00		1.00		1.00		1.00		1.00	
Mid	1.18***	(1.07–1.29)	1.00	(0.90–1.11)	1.04	(0.92–1.18)	1.10*	(1.02–1.19)	1.15**	(1.05–1.25)	1.33***	(1.19–1.48)	0.97	(0.81–1.16)	1.01	(0.93–1.12)	1.00	(0.91–1.10)
Low	1.21***	(1.10–1.34)	0.97	(0.86–1.09)	1.20**	(1.06–1.37)	1.18***	(1.08–1.29)	1.11*	(1.01–1.23)	1.38***	(1.23–1.56)	0.91	(0.74–1.12)	0.88*	(0.78–0.99)	1.01	(0.91–1.13)
Incorporated cultural capital																		
High	1.00		1.00		1.00		1.00		1.00		1.00		1.00		1.00		1.00	
Mid	1.25***	(1.13–1.39)	1.25***	(1.12–1.39)	1.15*	(1.01–1.31)	1.14**	(1.05–1.24)	1.26***	(1.14–1.40)	1.33***	(1.17–1.51)	1.42***	(1.16–1.73)	1.11*	(1.01–1.23)	1.18**	(1.06–1.31)
Low	1.35***	(1.22–1.51)	1.18**	(1.05–1.33)	1.14	(0.99–1.31)	1.30***	(1.19–1.42)	1.52***	(1.37–1.68)	1.74***	(1.53–1.98)	1.76***	(1.44–2.14)	1.06	(0.95–1.19)	1.37***	(1.23–1.52)
Total social capital																		
High	1.00		1.00		1.00		1.00		1.00		1.00		1.00		1.00		1.00	
Mid	1.03	(0.93–1.13)	0.99	(0.90–1.10)	0.99	(0.87–1.13)	1.04	(0.96–1.13)	0.98	(0.89–1.07)	1.08	(0.96–1.21)	0.94	(0.79–1.12)	1.03	(0.94–1.13)	1.00	(0.91–1.09)
Low	1.17***	(1.06–1.28)	1.04	(0.94–1.16)	1.27***	(1.12–1.44)	1.16***	(1.07–1.26)	1.08	(0.99–1.18)	1.30***	(1.16–1.46)	0.93	(0.77–1.12)	0.89**	(0.80–0.99)	1.05	(0.96–1.16)
Total economic capital																		
High	1.00		1.00		1.00		1.00		1.00		1.00		1.00		1.00		1.00	
Mid	1.00	(0.90–1.10)	1.01	(0.91–1.12)	1.12	(0.99–1.28)	0.99	(0.91–1.07)	0.96	(0.87–1.06)	0.96	(0.85–1.08)	1.03	(0.86–1.24)	0.90*	(0.81–0.99)	1.02	(0.93–1.12)
Low	1.13*	(1.02–1.25)	0.99	(0.88–1.12)	1.16*	(1.00–1.34)	1.10*	(1.01–1.20)	1.06	(0.95–1.17)	1.05	(0.93–1.19)	1.07	(0.87–1.31)	0.85**	(0.76–0.95)	0.98	(0.88–1.09)

* = *p* < .050, ** = *p* ≤ .010, *** = *p* ≤ .001; *OR* odds ratio. ^a^ All models included education and the following confounders: age, sex, ethnic background and marital status

^b^Varying sample sizes due to missing values on the food outcomes

In univariate models (as presented in Table 3), lower levels of cultural, social and economic capital were in general related to lower healthy food consumption and higher unhealthy food consumption (except for red meat). Incorporated cultural capital was most consistently and strongest associated with the outcomes, compared to institutionalised and objectivised cultural capital. Those with low social capital were more likely to report a low overall healthy food consumption (PR 1.17, 95% CI 1.06–1.28), and a low consumption of whole wheat bread (PR 1.27, 95% CI 1.12–1.44), fruit (PR 1.16, 95% CI 1.07–1.26), and meat-substitutes and fish (PR 1.30, 95% CI 1.16–1.46). A low level of economic capital was associated with low overall healthy food consumption, low whole-wheat bread consumption, low fruit consumption, and low red meat consumption.

In multivariate models including educational level, and cultural, social and economic capital, cultural capital remained significantly associated with all outcomes (Table 4). Having less cultural capital was related to a lower overall healthy food consumption (PR 1.35, 95% CI 1.22–1.49), lower consumption of whole wheat bread (PR 1.21, 95% CI 1.05–1.38), vegetables (PR 1.55, 95% CI 1.40–1.71), and meat-substitutes & fish (PR 1.74, 95% CI 1.53–1.97), and a higher consumption of fried food (PR 1.59, 95% CI 1.31–1.93). Social capital remained associated with overall healthy food consumption, whole wheat bread consumption, and meat-substitutes & fish consumption, but economic capital with none of the outcomes. In these multivariate models, PR’s for the low educational group attenuated considerably after inclusion of the capital variables (Table 4), compared to the model with only confounders (Table 3). For instance, the PR of the lowest compared to the highest educational group for having a low overall healthy food consumption decreased from 1.48 (95% CI 1.28–1.73) (when only adjusted for confounders; Table 3) to 1.22 (95% CI 1.04–1.43), when cultural, social and economic capital were taken into account (Table 4). However, educational inequalities in food consumption remained significant for all outcomes.

**Table 4:** Simultaneous adjustment of total cultural, social and economic capital on educational inequalities in food consumption, adjusted for confounders^a^

	Low overall healthy food consumption		Low whole wheat bread		Low vegetable		Low fish & meat-substitutes		High fried food	
	*(n = 2947)* ^b^		*(n = 2900)* ^b^		*(n = 2934)* ^b^		*(n = 2833)* ^b^		*(n = 2853)* ^b^	
	OR	(95% CI)	OR	(95% CI)	OR	(95% CI)	OR	(95% CI)	OR	(95% CI)
Educational level										
1 High	1.00		1.00		1.00		1.00		1.00	
2	1.09	(0.98–1.20)	1.23**	(1.08–1.40)	1.20***	(1.09–1.32)	1.18**	(1.05–1.33)	1.42***	(1.18–1.71)
3	1.08	(0.97–1.20)	1.26**	(1.09–1.47)	1.18**	(1.06–1.31)	1.10	(0.97–1.26)	1.35**	(1.08–1.69)
4 Low	1.22*	(1.04–1.43)	1.19	(0.92–1.53)	1.17	(0.98–1.40)	1.23*	(1.00–1.51)	1.66**	(1.15–2.38)
Total cultural capital										
High	1.00		1.00		1.00		1.00		1.00	
Mid	1.23***	(1.11–1.37)	1.13	(0.98–1.30)	1.30***	(1.16–1.45)	1.32***	(1.15–1.51)	1.29*	(1.05–1.59)
Low	1.35***	(1.22–1.49)	1.21**	(1.05–1.38)	1.55***	(1.40–1.71)	1.74***	(1.53–1.97)	1.59***	(1.31–1.93)
Total social capital										
High	1.00		1.00		1.00		1.00		1.00	
Mid	1.01	(0.91–1.11)	0.98	(0.86–1.11)	0.96	(0.87–1.05)	1.05	(0.94–1.18)	0.92	(0.77–1.10)
Low	1.11*	(1.01–1.22)	1.23**	(1.08–1.40)	1.02	(0.93–1.12)	1.21***	(1.08–1.36)	0.87	(0.72–1.05)
Total economic capital										
High	1.00		1.00		1.00		1.00		1.00	
Mid	0.97	(0.87–1.07)	1.09	(0.95–1.24)	0.93	(0.84–1.02)	0.89	(0.79–1.01)	1.00	(0.83–1.21)
Low	1.06	(0.95–1.18)	1.08	(0.93–1.25)	0.98	(0.89–1.09)	0.93	(0.82–1.06)	1.03	(0.83–1.27)

* = *p* < .050, ** = *p* ≤ .010, *** = *p* ≤ .001; *OR* odds ratio. ^a^ All models included educational level, total cultural capital, total social capital, total economic capital, and confounders (age, sex, ethnic background and marital status). ^b^ Varying sample sizes due to missing values on the food outcomes

## Discussion

This is the first study to investigate the contributions of cultural, social and economic capital to educational inequalities in food consumption among adults. Educational inequalities in healthy food consumption were larger than those in unhealthy food consumption. Cultural capital contributed to the explanation of educational inequalities in food consumption more so than social and economic capital. Associations between cultural capital and food consumption remained significant when adjusted for social and economic capital.

Our finding that low educational groups consumed less healthy foods is in line with previous empirical studies [1–5]. These results also largely confirm Bourdieu’s own observations regarding food consumption, as reported in his book *Distinction: A Social Critique of the Judgement of Taste* [21]. He wrote that individuals from lower classes with a low level of capital tended to prefer ‘heavy, fatty, fattening foods, which are also cheap’ ([21], p. 177) and preferred ‘the plentiful’ as opposed to ‘the light, refined, and delicate foods’ valued by high classes with higher levels of capital [19, 21]. Further, he observed that those with high cultural capital seemed to be more inclined towards asceticism and pursue original foods with an abundance of vegetables, whereas those with high economic capital preferred more traditional, rich dishes - a taste that resembles those of lower classes [19, 21]. In line with this, we saw that those with higher economic capital were more likely to consume more of the “traditional, rich” red meat products (e.g. beef, pork, mince, and burgers).

While we found clear positive relations between educational level and healthy food consumption, and between cultural capital and healthy food consumption, fewer associations with unhealthy food consumption were found. Apparently, possessing higher levels of cultural capital facilitates the choice of healthy foods, but having more cultural capital does not seem to prevent against unhealthy food consumption. This finding may suggest that high educated, with more cultural capital, make healthy food choices for *other* reasons than for reasons of health (because, if the latter was the case, one would expect them to also refrain from unhealthy foods). Following Bourdieu’s line of reasoning, one interpretation could be that healthy foods are consumed for reasons of distinction, and that consuming healthy foods is considered a more effective means of ‘distinction’ than refraining from unhealthy foods. A reason for this could be that a behaviour you practise (e.g. eating healthy foods) is more visible and obvious, and therefore more useful for distinction, than a behaviour you refrain from (e.g. not eating unhealthy foods). The findings from a study into educational differences in ‘super foods’ consumption also point to this mechanism of distinction [39].

Economic capital showed only weak associations with food consumption, and did hardly contribute to the explanation of educational inequalities in healthy and unhealthy food consumption. This could be due to the selection of the specific food products that were analysed, as the unhealthy food options (white bread, fried foods, red meat products) may not necessarily be cheaper than their healthy counterparts. However, also previous research from the Netherlands did not found indications that price considerations are an important barrier for healthy food consumption among low educational groups in the Netherlands [2, 40, 41].

Our finding that cultural capital adds to explanation of educational inequalities in food choices, over and above economic and social capital, is in line with two previous studies among adolescents [18, 20]. Taking all capital variables into account in multivariate models considerably reduced the educational inequalities in healthy and unhealthy food consumption, but not completely. This indicates that other factors than those covered by cultural, social, and economic capital contribute to the observed gradients. A factor that we did not take into account, and that has found to play a role in the international context (e.g. U.K. and U.S.), is the neighbourhood food environment, i.e. the accessibility and availability of healthy foods [42, 43]. In a compact country like the Netherlands, where the average distance from home to a supermarket (in which, in general, a wide variety of healthy, good-quality food products is available against reasonable prices) is 900 m [44], we have no signs of the existence of so-called ‘food deserts’ [45], nor the existence of large inequalities in food environmental attributes between low and high socioeconomic neighbourhoods [46].

### Methodological considerations

This first large-scale study investigating cultural, social and economic capital in order to quantify their role for explaining educational inequalities in healthy and unhealthy food consumption among adults has some clear strengths. We operationalised all capital variables in a theory-based way, developed indicators for cultural capital that may be more likely causally related to healthy food consumption than the more classical indicators (e.g. number of books, cultural participation [19, 28]), the sample was large with almost 3000 respondents, and multiple outcomes of food consumption were investigated. However, also limitations need to be taken into account when interpreting the results. A first limitation is the measurement of food consumption, which only provided frequency information of food products consumed, not portion sizes. Clearly such a questionnaire can only provide crude estimates of food consumption, and does not allow to calculate whether participants meet recommendations for certain intakes, e.g. fruits and vegetables, nor to calculate a score indicating the overall healthiness of a person’s diet. Therefore, this study cannot provide evidence that having more cultural capital leads to an overall more healthy diet – something that should be investigated in future research. However, analysing specific food groups as separate outcomes also has advantages. First, it allowed us to investigate to what extent certain types of capitals are more or less important for some food outcomes than for others. Secondly, this approach showed that educational inequalities are more pronounced for healthy than unhealthy food outcomes – something that would not have become clear from analysing an overall diet score.

Secondly, the measures of cultural capital were developed in a systematic way [5], however, these were framed specifically in relation to food consumption. Being more proximal to the outcome of interest may have contributed to the stronger associations of cultural capital with food consumption, compared to the more generally-framed economic and social capital measures. Thirdly, the inequalities in food consumption we report are likely an underestimation of the true inequalities, for two reasons: 1) replacement of missing values on the educational level variable in the GLOBE-2011 survey data with information from the GLOBE-2004 survey may have introduced a bias, as participants’ highest attainted educational level could have increased over time, and 2) the response to the GLOBE-2011 was relatively good (67.2%), but lower among low educated (55.5%). Lastly, this cross-sectional study cannot show insight in the direction of the associations between educational level, capital and food consumption. Acknowledging these limitations, the paper represents a novel contribution to the existing literature on educational inequalities in food consumption.

### Recommendations for policy and future research

Cultural capital offers new entry-points for the promotion of healthy food consumption among low educational groups. The strong association between cultural capital and healthy food consumption implies that deeply rooted cultural resources acquired over a lifelong socialization period are relevant for food consumption. In order to improve healthy food consumption it may be important to start early in life and make healthy diets part of this socialization process, in order to, for instance, develop the broad range of skills needed for a healthy diet. Future research should investigate the specific (and causal) underlying mechanisms between educational level, cultural capital and healthy food consumption, which is needed for the development of evidence-based interventions. Especially, a better understanding is needed in the socio-cultural processes through which cultural capital is acquired, and qualitative studies are likely necessary to gain such insights. Recent work from our group (Oude Groeniger J, de Koster W, van der Waal J, Mackenbach JP, Kamphuis CBM, van Lenthe FJ: How does cultural capital make you thin? Exploring cultural signifiers that explain the relationship between cultural capital and body mass index, submitted) points to the importance of cultural signifiers (i.e. asceticism, refinement, reflexivity) as mechanisms between cultural capital and maintaining a healthy weight.

## Conclusion

Cultural capital is related to healthy food consumption and contributes to the explanation of educational inequalities in healthy food consumption, over and above economic and social capital. The socialisation processes through which cultural capital is acquired could offer new entry-points for the promotion of healthy food consumption among low educational groups.

## Additional files

Additional file 1: Table A Means and standard deviations (SD) for the daily frequency scores of the food consumption outcomes, by educational level. Table B Prevalences of high/low scores on the food outcomes for the total sample, and within the groups with high and low overall healthy food consumption. (PDF 35 kb)

Additional file 2: Measurement and construction of variables for cultural, social, and economic capital. (PDF 61 kb)
