# Supplementary material for: Does cultural capital contribute to educational inequalities in food consumption in the Netherlands? A cross-sectional analysis of the GLOBE-2011 survey
Source: Int J Equity Health. 2018 Nov 15;17:168. doi: 10.1186/s12939-018-0884-z (PMC6238371; doi:10.1186/s12939-018-0884-z)
Supplement: Supplementary file 1 — Table A Means and standard deviations (SD) for the daily frequency scores of the food consumption outcomes, by educational level. Table B Prevalences of high/low scores on the food outcomes for the total sample, and within the groups with high and low overall healthy food consumption. (PDF 35 kb) [file 12939_2018_884_MOESM1_ESM.pdf]

Web-appendix 2.

Table A. Means and standard deviations (SD) for the daily frequency scores[a] of the food consumption outcomes, by educational level.

Educational level		Overall healthy food consumption	Overall unhealthy food consumption	Whole wheat bread	Fruit	Vegetables	Fish/meat substitute	Fried food	Red meat	Soft drink
1,00 - Low	Mean	3,3782	1,3786	,7781	,6914	,9766	,1570	,1072	,4136	,1984
	SD	1,33754	,87949	,35511	,36660	,46608	,19488	,09073	,31014	,30594
2,00	Mean	3,7925	1,3644	,8104	,7203	1,1004	,1597	,1032	,3855	,1626
	SD	1,09753	,80118	,32351	,34548	,36389	,15061	,06476	,26018	,28450
3,00	Mean	3,6207	1,4393	,7660	,6684	1,0869	,1851	,1136	,4023	,1706
	SD	1,16095	,78883	,35175	,33913	,36886	,17858	,08219	,23945	,27292
4,00 - High	Mean	3,8999	1,4079	,8267	,7003	1,1941	,2180	,0970	,3925	,1560
	SD	1,05413	,73387	,30588	,33039	,38325	,18997	,08143	,22831	,25831
Total	Mean	3,7594	1,4007	,8026	,6976	1,1229	,1876	,1040	,3942	,1642
	SD	1,12519	,77983	,32784	,34010	,38365	,17744	,07761	,24709	,27330

[a] Daily frequency scores were calculated as follows. In the food frequency questionnaire, participants indicated the number of days per week each food product was consumed. This number was converted to an indicator for 'average daily frequency' by the following formula (Pollard et al. *J Biosoc Sci*. 1998 Apr;30(2):165-79): never: 0; less than once a week: 0.10; 1-2 days per week: 0.20; 3-4 days per week: 0.50; 5-6 days per week: 0.80; every day: 1. The scores for 'overall healthy food consumption' and 'overall unhealthy food consumption' are higher than 1, as these are the sum of daily frequency scores of multiple food products. An 'overall healthy food consumption' score was constructed as the sum of the consumption of fruit, cooked vegetables, raw vegetables, whole wheat bread, skimmed milk, low fat cheese, chicken, fish, and meat-substitutes (like tofu). To calculate this score, the average daily frequencies (ranging from 0 till 1, as detailed above) for each of these products were summed. Similarly, an 'overall unhealthy food consumption' score was constructed as the sum of the frequencies of consumption of fried food, candy, white bread, soft drinks, whole milk, high fat cheese, and red meat (beef, pork, lamb, mince, and burgers).

Table B. Prevalences of high/low scores on the food outcomes for the total sample, and within the groups with high and low overall healthy food consumption.

		Total (%)	Overall healthy food consumption	
			High (%)	Low (%)
Overall unhealthy food consumption	High	47.7	41.3	53.6
	Low	52.3	58.7	46.4
Whole wheat bread	High	63.4	81.4	46.3
	Low	36.6	18.6	53.7
Fruit	High	43.4	64.9	23.0
	Low	56.6	35.1	77.0
Vegetables	High	47.5	66.8	29.3
	Low	52.5	33.2	70.7
Fish/meat substitute	High	55.7	65.6	46.4
	Low	44.3	34.4	53.6
Fried food	High	21.5	16.9	25.7
	Low	78.5	83.1	74.3
Red meat	High	50.3	49.9	50.6
	Low	49.7	50.1	49.4
Soft drink	High	50.4	42.7	57.6
	Low	49.6	57.3	42.4
