# Supplementary material for: Does cultural capital contribute to educational inequalities in food consumption in the Netherlands? A cross-sectional analysis of the GLOBE-2011 survey
Source: Int J Equity Health. 2018 Nov 15;17:168. doi: 10.1186/s12939-018-0884-z (PMC6238371; doi:10.1186/s12939-018-0884-z)
Supplement: Supplementary file 2 — Measurement and construction of variables for cultural, social, and economic capital. (PDF 61 kb) [file 12939_2018_884_MOESM2_ESM.pdf]

Web-Appendix 1 – Measurement and construction of variables for cultural, social, and economic capital

In this appendix, the survey questions that were used to measure cultural, social and economic capital are detailed (see Table A below). In the text, the questionnaire items are described and the construction of the variables for the analyses is explained. At the end of this appendix, a reference list is included with the sources on which the questionnaire items were based.

Cultural capital

Questions to measure institutionalised, objectivised and incorporated cultural capital were based on often used indicators for cultural capital that came forth from a systematic review into existing measures (Kamphuis et al, 2015).

Family institutionalised cultural capital was operationalised by educational level of the respondent's father, mother and partner (each with four categories, i.e. 1= no education or primary education; 2= lower vocational and intermediate general education; 3= intermediate vocational and higher general education; and 4= higher professional education and university).[1] The Cronbach's alpha for these three items was .762. We calculated the mean score of these three items as overall measure of family institutionalised cultural capital. For n=713 respondents, this mean value was based on less than three items, due to missing values. Respondents with missing values on all three items (n=394) received a missing value for family institutionalised cultural capital. Two variables for *family institutionalised cultural capital* were created: one variable by dividing the mean score into quintiles with 1= low and 5= high (which was used to compute total cultural capital, see below), and a second variable by dividing the mean score into tertiles with 1= low and 3= high (which was used in further analyse, as presented in Tables 2 and 3).

Objectivised cultural capital was consistently measured in the literature by cultural possessions[1] and we translated this to a list of possessions related to food choice behaviour. We asked respondents whether they owned several cooking-related possessions, i.e. a stove, cook book(s), set of knives, kitchen scale, and juicer (yes/no) (Cronbach's alpha: .545). A sum score for objectivised cultural capital was created. Two variables for o*bjectivised cultural capital* were created: one variable by dividing the mean score into quintiles with 1= low and 5= high (which was used to compute total cultural capital, see below), and a second variable by dividing the mean score into tertiles with 1= low and 3= high (which was used in further analyse, as presented in Tables 2 and 3).

From the measures of *incorporated cultural capital* that came forth from the literature review, we selected the main underlying themes, i.e. participation in cultural activities, skills, and knowledge, and searched the literature to find existing questionnaires to measure these in relation to food choices. [1] *Participation* was measured with two items, for which participants could indicate a frequency, namely: "In the last month, how many times have you met with people in a public place to have some

food?" (Grootaert 2004), and "In the last month, how many times have people visited you in your home to have dinner, or have you visited people for dinner in their home?".[2] Both frequencies were summed in one variables, and the sum was divided in quintiles, with 1= low and 5= high food-related participation. For food choice-related skills we distinguished three types: *cooking skills* [3-4], *grocery shopping skills*,[5] and *skills to find and process information* about nutrients and food preparation (adapted from Chew et al. (2004)[6]). We created variables for each group of skills, based on multiple items from the questionnaire (Kamphuis et al., 2015). Variables were divided in quintiles, with 1= low skill level, vs. 5= high skill level. *Nutrition knowledge* was measured with an existing questionnaire including 16 items (Cronbach's alpha: .519), namely four different questions (e.g. Do these products contain high or low levels of added sugar? Do these products contain high or low levels of protein?), that were asked with regard to four products each (e.g. bananas, chicken, chocolate, red meat) (three answer categories: high, low, don't know).[7-8] A sum score of all correct answers was made (ranging from 0-16), and divided in quintiles, with 1=low and 5=high nutrition knowledge. *Total incorporated cultural capital* was measured by the mean score of the participation, skills and knowledge variables. Two variables for *total incorporated cultural capital* were created: one variable by dividing the mean score into quintiles with 1= low and 5= high (which was used to compute total cultural capital, see below), and a second variable by dividing the mean score into tertiles with 1= low and 3= high (which was used in further analyses, as presented in Tables 2 and 3).

Total cultural capital was created by computing the mean score of the quintile-variables for family institutionalised, objectivised, and total incorporated cultural capital, which was grouped into tertiles, with 1= low total cultural capital, and 3= high total cultural capital.

Social capital

Social capital is seen as a multidimensional concept, although there is little consensus on its measurement. In our survey, we selected commonly used indicators to measure six dimensions of social capital: social support, health-related social leverage, interpersonal relationship network, social participation, perceptions of trust, and perceived neighbourhood social capital.[9]

Social support was measured with 9 items.[10] Respondents were asked how often they could turn to someone, e.g. for having a nice day out, for love and affection, for advice, etc. (answers a 5-point Likert scale ranging from always to never). Based on these 9 items, a mean score was created, which was divided in quintiles with 1=low social support, 5=high social support.

Health-related social leverage was measured with questions asking the respondents to whom they could turn for advice [11] about five health-related topics (more than one answering option could be ticked: nobody, partner/family member, friend, colleague, acquaintance), e.g. to lose weight, to get more physical activity. For each of the five topics, a sum score was calculated of the total number of people that could potentially help. These five sum scores were summed and divided by 5, to calculate

the overall mean score for social leverage. This score was divided in quintiles with 1=low, and 5=high health-related social leverage.

The size of the respondent's *interpersonal relationship network* was measured with one item asking respondents to indicate the number of good friends and family members (including their partner) they have. The answers to this open question ranged from 0 to 100, with a median of 10.0, which were divided in quintiles with 1=small, and 5=large interpersonal relationship network.

Social participation was measured with a question asking respondents to indicate, for six social organisations (e.g. sports club, political party, church), whether they were involved in such an organisation (e.g. by being a member, attending meetings, doing voluntary work). [2] Answers to these six items were summed, and divided into quintiles with 1=low, and 5=high social participation.

Perceptions of trust were measured with three standard items [12] on a 10-point Likert scale, e.g. "Generally speaking, would you say that most people can be trusted, or that you can't be too careful in dealing with people?" (You can't be too careful – Most people can be trusted), "Do you think that most people would try to take advantage of you if they got the chance, or would they try to be fair?" (Most people would try to take advantage of me – Most people would try to be fair), "Would you say that most people deserve your trust or that only very few deserve your trust?" (Very few people deserve my trust - Most people deserve my trust). The mean scores on these three items ranged from 1 to 10, with a median of 7.3, and were divided in quintiles with 1= low, and 5= high general trust.

Perceived neighbourhood social capital was measured with four commonly-used statements, e.g. "People in this neighbourhood are willing to help each other"; "If I get the chance, I move out of this neighbourhood" (with answers on a 5-point Likert scale, from 1= totally disagree, to 5= totally agree).[13] The four items were recoded in such a way that a higher score meant: more positive perception of neighbourhood social capital. A mean score was created based on these items, and divided in quintiles with 1=low neighbourhood social capital, and 5=high neighbourhood social capital.

Total social capital was created by computing the mean score of social support, health-related social leverage, interpersonal relationship network, social participation, perceptions of trust, and neighbourhood social capital, which was divided into tertiles, with 1= low social capital, and 3= high social capital.

Economic capital

Four indicators of economic capital were measured in our survey: household equivalent income, home ownership, crowding, and financial strain.[14] *Household equivalent income* was calculated by dividing the total household income per month by the square-root of the number of people living from this income. This variable was subsequently divided into quintiles, with 1= low, and 5= high household equivalent income. *Home ownership* was categorised as, 1= renter, 5= homeowner.

Crowding was calculated as the number of rooms in the house, divided by the number of persons living in the household, and divided in quintiles with 1=high, and 5=low crowding. *Financial strain* was measured with one question asking whether respondents had experienced any difficulties in paying bills, e.g. for food, rent, and electricity, during the preceding year (response categories: 'no difficulties', 'some difficulties' and 'big difficulties'). This variable was divided into 1= at least some financial strain, 5= no financial strain.

Total economic capital was created by computing the mean score of the variables for household equivalent income, crowding, homeownership, and financial strain, which was divided into tertiles with 1= low economic capital and 3= high economic capital.

Table A. Questionnaire items used to measure cultural capital (related to food choices), social capital and economic capital

Variables	Questionnaire items	Answering categories	Adapted from existing questionnaire (reference)
Family institutionalised cultural capital			
Highest educational credentials of the respondent's father, mother, and (if applicable) partner	(3 items) Please indicate the highest level of education that has been achieved by: a) your father, b) your mother, c) your partner.	1 = No education or primary education; 2= Lower vocational education or higher general secondary education;<br>3 = Intermediate vocational education or higher general secondary education;<br>4= higher professional education or university;	[1]
Objectivised cultural capital			
Cooking equipment	(5 items) Could you please indicate whether you own the following cooking objects? a) Oven, b) Cookery book(s), c) Set of knives , d) Kitchen scales, e) Fruit juicer	Yes, no.	-
Incorporated cultural capital			
Participation	(2 items) a)"In the last month, how many times have you met with people in a public place to have some food?", b) "In the last month, how many times have people visited you in your home to have dinner, or have you visited people for dinner in their home?"	Open question	[2]
Cooking skills	(3 items) Below you may find three statements about cooking. Please indicate for each of the following statements whether you agree or disagree. a) I know several ways to prepare fish. b) I can prepare a lot of meals even without a recipe. c) I know several ways to prepare vegetables.	Answers on a 5-point Likert scale, from 1= Totally disagree, to 5= Totally agree; and 'Don't know'.	[3-4]

Grocery shopping skills	(2 items) Below you may find two statements about grocery shopping. Please indicate for both statements how often this applies to you. a) Before I go shopping for food, I make a list of everything I need. b) Usually I do not decide what to buy until I am in the shop.	Always; Usually; Sometimes; Seldom; Never.	[5]
Food information skills	(4 items) Below are some questions about food information. Please indicate for each question how often this applies to you. A) Do you read the nutrition information and information about ingredients on food packages? b) Do you use the information about nutritional value on food packages to decide what foods you buy? c) Do you look up information about foodstuffs on the internet? (For instance on the website of the Nutrition information centre?) d) Do you use recipes from cookery books, from the internet, or from magazines?	Always; Usually; Sometimes; Seldom; Never.	[6]
Nutrition knowledge	(16 items) Please indicate for the following four food items whether they are high or low in added sugar: a) Bananas, b) Unflavoured yoghurt, c) Ice-cream, d)Tomato ketchup.<br>Please indicate for the following four food items whether they are high or low in protein: a) Chicken, b) Cheese, c) Fruit, d)Broccoli.<br>Please indicate for the following four food items whether they are high or low in fibre. a) Eggs, b) Nuts, c) Chicken, d)Broccoli.<br>Please indicate for the following four food items whether they are high or low in saturated fat? a) Olive oil, b) Nuts, c)Red meat (pork, mutton), d)Chocolate	High; Low; Don’t know	[7-8]
Social capital			
Social support	(9 items) Sometimes people need other people as company, for advice, or help. Could you indicate for each of the following types of support how often this is available to you, if you need it? a) Someone to give you love and affection; b) Someone to have a nice day out	Always; Usually; Sometimes; Seldom; Never.	[10]

	with; c) Someone you trust, to talk about personal problems; d) Someone to spend leisure time with; e) Someone who cooks for you if you cannot do that yourself; f) Someone who cares for you if you are ill; g) Someone with whom you can share your most personal worries and fears; h) Someone that gives you advice how to handle personal problems; i) Someone who loves you and gives you the feeling that you are a valuable person.		
Health-related social leverage	(5 items) If you would need advice or help with one of the following topics, would you have someone you could easily turn to? (you may tick more than one box)<br>Do you know someone to turn to if you would want:<br>a) to lose weight?<br>b) to be more physically active?<br>c) to quit smoking?<br>d) to consume fewer alcoholic drinks?<br>e) medical advice, in case you are not satisfied with your doctor?	Nobody, partner/family member, friend, colleague, acquaintance (more than one answer could be ticked)	[11]
Size of interpersonal relationship network	(1 item) How many good friends and close family members do you have?	Open question	
Social participation	(6 items) With which of the following organisations do you feel involved? (which means that you are a member, or attending meetings, or do voluntary work) You may tick more than one answer.	Sports club; leisure association; trade union; political party; church; neighbourhood association; none of these organisations; another organisation, namely…	[2]
Perceptions of trust	(3 items) a) Generally speaking, would you say that most people can be trusted, or that you can't be too careful in dealing with people?"; b) "Do you think that most people would try to take advantage of you if they got the chance, or would they try to be fair?"; c) "Would you say that most people deserve your trust or that only very few deserve your trust?".	Answers on a 10-point Likert scale ranging, per item, from:<br>a) You can't be too careful – Most people can be trusted<br>b) Most people would try to take advantage of me – Most people would try to be fair<br>c) Very few people deserve my trust - Most people deserve my trust	[12]

Perceived neighbourhood social capital	(4 items) Below you may find four statements about people living in your neighbourhood. Please indicate to what extent you agree with each statement: a) I often feel lonely in this neighbourhood; b) If I get the chance, I move out of this neighbourhood; c) People in this neighbourhood treat each other well; d) People in this neighbourhood are willing to help each other.	Answers on a 5-point Likert scale, from 1= Totally disagree, to 5= Totally agree	[13]
Economic capital			
Household equivalent income	(2 items) a) Could you indicate your total net household income per month?<br>b) How many people in total (in and outside your household) live from this income?<br>Household equivalent income was calculated by the square-root of the number of people living from this income.	a) about 0-1200 euro per month; 1200-1800 euro per month; 1800-2600 euro per month; 2600-4000 euro per month; more than 4000 euro per month; I don't know, or I don't want to tell.<br>b) Open question	[14]
Home ownership	(1 item) Are you a home owner, or do you rent a house?	renter, home owner	[14]
Crowding	(2 items) How many people in total live in your house? (including yourself)<br>How many rooms has your house? (do <u>not</u> count the garage, basement, kitchen, toilet, bathroom)<br>Crowding was calculated by number of rooms in the house, divided by the number of persons living in the household.	Open questions.	[14]
Financial strain	(1 item) Did you experience any difficulties in paying bills last year, e.g. for food, rent, and electricity?	No difficulties; some difficulties; big difficulties.	[14]

References

1 Kamphuis CBM, Jansen T, Mackenbach JP, et al. Bourdieu's Cultural Capital in Relation to Food Choices: A Systematic Review of Cultural Capital Indicators and an Empirical Proof of Concept. PLoS ONE 2015;10(8): e0130695.

2 Grootaert C, Narayan D, Jones VN, Woolcock M (2004) Measuring social capital; an integrated questionnaire. Washingon: The World Bank.

3 Caraher M, Dixon P, Lang T, Carr-Hill R (1999) The state of cooking in England: the relationship of cooking skills to food choice. British Food Journal 101: 590–609.

4 Horst van der K, Brunner TA, Siegrist M (2011) Ready-meal consumption: associations with weight status and cooking skills. Public Health Nutr 14: 239–245.

5 Scholderer J, Brunsø K, Bredahl L, Grunert KG (2004) Cross-cultural validity of the food-related lifestyles instrument (FRL) within Western Europe. Appetite 42: 197–211. PMID: 15010184

6 Chew LD, Bradley KA, Boyko EJ (2004) Brief questions to identify patients with inadequate health literacy. Fam Med 36: 588–594.

7 Parmenter K, Wardle J (1999) Development of a general nutrition knowledge questionnaire for adults. Eur J Clin Nutr 53: 298-308.

8 Wardle J, Parmenter K, Waller J (2000) Nutrition knowledge and food intake. Appetite 34: 269–275.

9 Sun X, Rehnberg C, Meng Q. How are individual-level social capital and poverty associated with health equity? A study from two Chinese cities. International Journal for Equity in Health 2009, 8:2 doi:10.1186/1475-9276-8-2

10 Sherbourne CD, Stewart AL. The MOS Social Support Survey. Soc Sci Med 1991;32(6):705-14

11 Van der Gaag MPJ, Snijders TAB. The Resource Generator: measurement of individual social capital with concrete items. *Social Networks* 2005;27:1-29.

12 European Social Survey, 2008, round 4
http://www.europeansocialsurvey.org/data/download.html?r=4 (accessed 27 December 2016)

13 Kamphuis CB, Van Lenthe FJ, Giskes K, Huisman M, Brug J, Mackenbach JP. Socioeconomic status, environmental and individual factors, and sports participation. *Med Sci Sports Exerc* 2008 Jan;40(1):71-81. doi: 10.1249/mss.0b013e318158e467.

14 van Oort FV, van Lenthe FJ, Mackenbach JP. Material, psychosocial, and behavioural factors in the explanation of educational inequalities in mortality in The Netherlands. *J Epidemiol Community Health* 2005 Mar;59(3):214-20.
